# Continuous positive airway pressure for respiratory support during COVID-19 pandemic: a frugal approach from bench to bedside

**DOI:** 10.1186/s13613-021-00828-2

**Published:** 2021-03-02

**Authors:** Guillaume Carteaux, Manuella Pons, François Morin, Samuel Tuffet, Arnaud Lesimple, Bilal Badat, Anne-Fleur Haudebourg, François Perier, Yvon Deplante, Constance Guillaud, Frédéric Schlemmer, Elena Fois, Nicolas Mongardon, Mehdi Khellaf, Karim Jaffal, Camille Deguillard, Philippe Grimbert, Raphaëlle Huguet, Keyvan Razazi, Nicolas de Prost, François Templier, François Beloncle, Alain Mercat, Laurent Brochard, Vincent Audard, Pascal Lim, Jean-Christophe Richard, Dominique Savary, Armand Mekontso Dessap

**Affiliations:** 1 Assistance Publique-Hôpitaux de Paris, CHU Henri Mondor-Albert Chenevier, Service de Médecine Intensive Réanimation, 51, Avenue du Maréchal de Lattre de Tassigny, 94010 Créteil Cedex, France; 2 Faculté de Santé, Groupe de Recherche Clinique CARMAS, Université Paris Est-Créteil, 94010 Créteil, France; 3 INSERM U955, Institut Mondor de Recherche Biomédicale, 94010 Créteil, France; 4 Département de Médecine d’Urgence, Faculté de Santé, Centre Hospitalier Universitaire d’Angers, Université d’Angers, Angers, France; 5 CNRS, INSERM 1083, MITOVASC, Université d’Angers, Angers, France; 6 Laboratoire Med2Lab ALMS, Antony, France; 7 Département d’Aval des Urgences, Assistance Publique-Hôpitaux de Paris, CHU Henri Mondor, 94010 Créteil, France; 8 Assistance Publique-Hôpitaux de Paris, CHU Henri Mondor, Unité de Pneumologie, 94010 Créteil, France; 9 Assistance Publique-Hôpitaux de Paris, CHU Henri Mondor, Unité des Maladies Génétiques du Globule Rouge, 94010 Créteil, France; 10 Assistance Publique-Hôpitaux de Paris, CHU Henri Mondor, Service d’Anesthésie-Réanimation Chirurgicale, 94010 Créteil, France; 11 Emergency Department, Assistance Publique-Hôpitaux de Paris, CHU Henri Mondor, 94010 Créteil, France; 12 Assistance Publique-Hôpitaux de Paris, CHU Henri Mondor, Service d’immunologie Clinique Et Maladies Infectieuses, 94010 Créteil, France; 13 Department of Cardiovascular Medicine, Assistance Publique-Hôpitaux de Paris, CHU Henri Mondor, 94010 Créteil, France; 14 Assistance Publique-Hôpitaux de Paris, CHU Henri Mondor, Service de Néphrologie et Transplantation, Centre de Référence Maladie Rare « Syndrome Néphrotique Idiopathique », 94010 Créteil, France; 15 Département de Médecine Intensive-Réanimation et Médecine Hyperbare, Faculté de Santé, Centre Hospitalier Universitaire d’Angers, Vent’ Lab, Université d’Angers, Angers, France; 16 Keenan Research Centre, Li Ka Shing Knowledge Institute, St. Michael’s Hospital, Toronto, Canada; 17 Interdepartmental Division of Critical Care Medicine, University of Toronto, Toronto, Canada; 18 Université Paris Est-Créteil, INSERM, IMRB, Equipe 21, 94010 Créteil, France; 19 Médecine Intensive Réanimation, CHU Grenoble Alpes, Grenoble, France; 20 INSERM, UMR 1066, Créteil, France; 21 IRSET (Institut de Recherche en Santé, environnement et travail)-UMR_S 1085, 49000 Angers, France

**Keywords:** COVID-19, Acute hypoxemic respiratory failure, Continuous positive airway pressure, Frugal innovation

## Abstract

**Background:**

We describe a frugal approach (focusing on needs, performance, and costs) to manage a massive influx of COVID-19 patients with acute hypoxemic respiratory failure (AHRF) using the Boussignac valve protected by a filter (“Filter Frugal CPAP”, FF-CPAP) in and out the ICU.

**Methods:**

(1) A bench study measured the impact of two filters with different mechanical properties on CPAP performances, and pressures were also measured in patients. (2) Non-ICU healthcare staff working in COVID-19 intermediate care units were trained with a video tutorial posted on a massive open online course. (3) A clinical study assessed the feasibility and safety of using FF-CPAP to maintain oxygenation and manage patients out of the ICU during a massive outbreak.

**Results:**

Bench assessments showed that adding a filter did not affect the effective pressure delivered to the patient. The resistive load induced by the filter variably increased the simulated patient’s work of breathing (6–34%) needed to sustain the tidal volume, depending on the filter’s resistance, respiratory mechanics and basal inspiratory effort. In patients, FF-CPAP achieved pressures similar to those obtained on the bench. The massive training tool provided precious information on the use of Boussignac FF-CPAP on COVID-19 patients. Then 85 COVID-19 patients with ICU admission criteria over a 1-month period were studied upon FF-CPAP initiation for AHRF. FF-CPAP significantly decreased respiratory rate and increased SpO_2_. Thirty-six (43%) patients presented with respiratory indications for intubation prior to FF-CPAP initiation, and 13 (36%) of them improved without intubation. Overall, 31 patients (36%) improved with FF-CPAP alone and 17 patients (20%) did not require ICU admission. Patients with a respiratory rate > 32 breaths/min upon FF-CPAP initiation had a higher cumulative probability of intubation (*p* < 0.001 by log-rank test).

**Conclusion:**

Adding a filter to the Boussignac valve does not affect the delivered pressure but may variably increase the resistive load depending on the filter used. Clinical assessment suggests that FF-CPAP is a frugal solution to provide a ventilatory support and improve oxygenation to numerous patients suffering from AHRF in the context of a massive outbreak.

**Supplementary Information:**

The online version contains supplementary material available at 10.1186/s13613-021-00828-2.

## Background

Frugal innovation is a process where needs and constraints are put forward in order to develop appropriate, adaptable, and affordable services and products [1]. This concept has proved its usefulness in intensive care units (ICUs) in low- and middle-income countries which often struggle with shortage of medication, devices and consumables, in addition to human and material resource limitations [1].

Unexpectedly, COVID-19 pandemic has imposed such challenges on all healthcare systems worldwide due to the massive influx of critically ill patients with acute hypoxemic respiratory failure (AHRF) [2]. Our hospital is located in one of the most affected areas in France [3].

In this context of a surge, ICU beds, ventilators and trained personnel can be lacking to manage a large number of patients almost simultaneously. In order to prepare for and address such a situation created by a massive influx of COVID-19 patients, we designed a strategy using a frugal solution (with focus on needs, optimized performance, and a substantial reduction in costs [1]) to safely administer CPAP as a bridge to intubation or as a prevention for intubation while minimizing the risks of aerosol dispersion. Boussignac CPAP (Vygon, Ecouen, France) is a cheap, easy-to-use, non-electrical device that works with no ventilator. However, as this “frugal CPAP” is an open system, an antimicrobial filter has to be inserted between the oro-nasal mask and the CPAP valve (“Filter Frugal CPAP”, FF-CPAP) to avoid viral aerosol dispersion. We hypothesized that the use of such FF-CPAP in intermediate care units (upstream ICU) may help manage large numbers of COVID-19 patients by better controlling their hypoxemia, enabling some patients to overcome the critical period and delaying ICU admission in others.

We therefore asked the following questions and herein report our translational, bench-to-bedside, approach:

1. Can an antimicrobial filter be added to a Boussignac valve without markedly deteriorating its performances? A bench study was first conducted to address this point, followed by physiological measurements in patients.
2. Does the simplicity of the system make it possible to train a large number of caregivers unfamiliar with this technique, with the constraint of social distancing and lack of trainers? We assessed the efficiency of an educative video posted on a massive open online course.
3. Was the use of the FF-CPAP feasible in conditions of a massive outbreak to maintain adequate levels of oxygenation and to manage patients out of the ICU? We retrospectively assessed the results obtained in 85 consecutive patients within 1 month.

## Methods

### Bench assessment

Full description of the Boussignac valve and FF-CPAP is provided in the Additional file 1. Briefly, the principle of FF-CPAP is to add a filter, which acts as a “microbiological barrier” between the oro-nasal mask and the CPAP valve. Numerous filters with a viral filtration efficiency above 99.99% are available. We conducted the entire bench assessment with two different filters characterized by different humidification and mechanical properties: the DAR™ Adult–Pediatric Electrostatic Filter HME Small (Hygrobac S; Covidien, Medtronic, Parkway, MN, USA) and the Clear-Guard™ (Intersurgical^®^, Fontenay Sous Bois, France). The first one (subsequently named “DAR filter”) is a heat and moisture exchanger with high humidification performances [4]; the second one (subsequently named “Clear-Guard filter”) is an electrostatic filter with poor humidification performances but lower resistance. The resistance of each filter was measured at the following air flow rates: 30, 60, 90 and 120 L/min.

#### Static measurements of airway pressure

The Boussignac CPAP valve was connected to the airway opening of a test lung model with or without the filter with anti-viral properties. To evaluate the impact of the filter on the effective pressure transmitted to the patient, we used a Michigan test lung (Michigan Instruments, Grand Rapids, USA), with a simulated compliance of 50 mL/cm H_2_O and two simulated resistances (5 and 15 cm H_2_O/L/s). The oxygen flow meter (ball flowmeter, 0–30 L/min, Technologie Biomedicale S.A.S, Noisy-Le-Sec, France) was adjusted to set the CPAP level at 6 and 10 cm H_2_O without the filter. The airway pressure measured inside the test lung was compared to the set CPAP level (measured with the dedicated manometer) without and with the filter placed in between.

#### Dynamic assessment of FF-CPAP

First, we assessed the end expiratory pressure and tidal volume generated by the FF-CPAP at different oxygen flow rates while simulating various inspiratory efforts. An oro-nasal mask (AcuCare non-vented mask, ResMed) was strapped to the face of a RespiSim^®^ Manikin (IngMar Medical, Pittsburg, PA, USA) and connected to a breathing simulator, Active Servo Lung 5000 (ASL5000^®^, IngMar Medical, Pittsburg, PA, USA; full methods in Additional file 1). The pressure into the oro-nasal mask was recorded at five constant oxygen flow rates: 10, 15, 20, 25, and 30 L/min while simulating four different inspiratory efforts (simulated inspiratory muscle pressures, Pmus): 5, 10, 15, and 20 cm H_2_O with the following respiratory mechanics: compliance = 50 mL/m H_2_O, resistance = 5 cm H_2_O/L/s.

Second, we assessed the impact of the additional resistive load related to the filter in dynamic conditions. The Boussignac valve was connected to the airway opening of the ASL 5000 lung simulator. The volume, airway pressure and Pmus were recorded without and with the filter in the following eight conditions: at two simulated effort (5 and 10 cm H_2_O of Pmus), two simulated resistances (5 and 15 cm H_2_O/L/s) with a constant compliance of 50 mL/cm H_2_O and two levels of CPAP (6 and 10 cm H_2_O). The decrease in volume (Delta Vt) induced by the filter and the maximum change in airway pressure between inspiration and expiration [expressed as peak-to-peak airway pressure (P-P)] were measured for each condition.

Dynamic pressure–volume loops were reconstructed based on volume and airway pressure recordings to calculate the work of breathing imposed by the device (WOBimposed, see Additional file 1 for more details). In each condition, the relative change in WOBimposed induced by the filter (ΔWOBimposed) was calculated and expressed as a percentage of the WOBimposed without the filter. Dynamic pressure–volume loops were also reconstructed based on volume and muscle pressure recordings, to calculate the theoretical increase in patient’s work of breathing required to maintain the tidal volume constant (see Additional file 1 for more details). Relative changes in patient’s work of breathing needed to maintain the tidal volume constant was calculated and expressed as a percentage of its value without the filter.

### Physiological measurements

In four patients receiving ventilatory support with the FF-CPAP with the DAR filter at four different oxygen flow rates (15, 20, 25, and 30 L/min), the pressure into the oro-nasal mask was recorded (see Additional file 1). A written informed consent was obtained from each patient and this physiological evaluation was approved by Mondor Institutional Review Board.

### Setup of intermediate care units and related training

The hospital admitted the first COVID-19 patient on February 15th, 2020. By March 14th, 2020, 52 patients were hospitalized, of whom 12 in ICU. Two intermediate care units (20 beds) with a 1/6 patient–nurse ratio were then created to treat patients with COVID-19 related acute hypoxemic respiratory failure (COVID-AHRF) who did not require immediate intubation.

Training program was rapidly programmed to enable non-ICU nurses and doctors to use FF-CPAP on COVID-AHRF patients. This training had to be continuous and at distance, hence the choice of a short (5 min) video tutorial (e-Video in the Additional file 2 or: http://www.reamondor.aphp.fr/covid19/). This tutorial was available on every computer in intermediate care units and integrated into a massive open online course (MOOC) dedicated to COVID-19 patients’ care (https://www.fun-mooc.fr/courses/course-v1:UPEC+169003+archiveouvert/about and https://covid19.coorpacademy.com/dashboard). The usefulness of this video tutorial was assessed retrospectively through a survey covering the medical and paramedical staff of the intermediate care units. We asked them to assess several statements (see Additional file 1) using a Likert scale model (strongly disagree/disagree/neutral/agree/strongly agree).

### Clinical study

This was a single-center retrospective study conducted in Henri Mondor University Hospital, Créteil, France, and approved by the institutional ethical committee of the French Intensive Care Society as a component of standard care. In accordance with French law, the patient's consent was waived, but each patient or his or her next of kin has been informed and given the opportunity to refuse the use of his or her personal data.

#### Patients

All consecutive patients with a “full code” order who received FF-CPAP as the first line ventilatory support for COVID-AHRF between March 14th and April 14th, 2020, were included. In case of “do not intubate” order upon FF-CPAP initiation, patients were not included. COVID-AHRF was defined as acute dyspnea (with a respiratory rate > 25 breaths/min and/or active contraction of accessory respiratory muscles), with escalating oxygen therapy ≥ 6 L/min with a non-rebreather facemask to maintain SpO_2_ > 90%, and new pulmonary infiltrates on chest X-rays [5] in a patient diagnosed with COVID-19. The latter was defined by a positive SARS-CoV-2 PCR on a naso-pharyngeal swab and/or a compatible computed tomography scan (CT-scan).

#### FF-CPAP therapy

FF-CPAP was assembled with the DAR filter and the same filter was left in place during the whole duration of FF-CPAP therapy. FF-CPAP support was initiated in patients with COVID-AHRF as defined above. FF-CPAP was not advised in case of hemodynamic instability or impaired neurologic status. The minimal oxygen flow rate with FF-CPAP was 15 L/min. The FF-CPAP was used in all cases in a continuous pattern interrupted to allow the patient to eat or whenever exceeded patient’s tolerance due to discomfort. During such interruptions, oxygen was supplied via the non-rebreather facemask. The presence of previously predefined respiratory criteria for intubation at the time of CPAP initiation was sought (intubation is recommended when at least two of such criteria are present) [6]: respiratory rate of > 40 breaths/min, signs of high respiratory muscle workload (meaning active contraction of accessory respiratory muscles), copious tracheal secretions, acidosis with pH < 7.35, and SpO_2_ < 90% for more than 5 min. Patients were intubated in case of persistence or emergence of signs necessitating intubation despite FF-CPAP therapy.

#### Outcomes

The main aim of the study was to assess the feasibility, efficiency and safety of using FF-CPAP to maintain adequate levels of oxygenation and to manage a massive influx of COVID-AHRF patients out of the ICU. Thus, we assessed the following main end points: (1) the effect of FF-CPAP on respiratory symptoms (decrease in respiratory rate) and oxygenation (increase in SpO_2_); (2) the duration of FF-CPAP therapy; (3) the proportion of patients who were ultimately not intubated, especially among patients exhibiting predefined criteria for intubation upon FF-CPAP initiation; (4) the proportion of patients remaining in intermediate care units without ICU admission; (5) the incidence of severe adverse event defined as hypoxemic cardiac arrest prior to intubation under FF-CPAP therapy; (6) potential factors associated with intubation in this population.

#### Data collection

We reviewed electronic medical records, laboratory and initial CT-scan findings for all patients. We collected data on age, sex, body mass index, medical history (smoking, chronic respiratory, cardiac, or kidney diseases, cancer), symptoms potentially related to COVID-19 (fever, cough, dyspnea, malaise, rhinorrhea, headache, vomiting, diarrhea, myalgia, and chest pain), and pre-hospitalization treatment (angiotensin-converting enzyme inhibitors, angiotensin II receptor blockers, corticosteroids, and non-steroidal anti-inflammatory drugs within the 7 days before hospital admission). Laboratory values at baseline were retrieved. Vital signs (respiratory rate, heart rate, mean blood pressure, oxygen flow rate) within the 24 h prior to FF-CPAP initiation as well as during the first hour of FF-CPAP therapy were collected. Duration of FF-CPAP delivery, the need for intubation, cardiac arrest prior to intubation and death within 28 days were also collected.

### Statistics

Data were analyzed using SPSS Base 20.0 statistical software package (SPSS, Chicago, IL, USA).

In the bench part of the study, normality of data’s distribution was verified using the Kolmogorov–Smirnov test of normality. Results were thus presented as means ± standard deviation. Comparisons between the conditions were performed using paired *t* test.

In the clinical assessment, no a priori sample size calculation was performed. The sample size was planned to correspond to the number of patients satisfying the inclusion criteria during the study period. Continuous data were expressed as medians (25th–75th percentiles) and compared using Mann–Whitney test for independent variables and Wilcoxon signed rank test for related variables. Categorical variables, expressed as percentages, were evaluated using Chi-square or Fisher exact tests as appropriate. The accuracy of respiratory rate measured before FF-CPAP initiation in detecting the need for intubation was assessed by receiver operating characteristic (ROC) curves. The threshold value of respiratory rate to predict intubation was then determined from analysis of ROC curves as the value that displayed the best compromise between sensitivity and specificity. Cumulative probability of intubation was evaluated using standard Kaplan–Meier actuarial techniques to estimate survival probability. Two-sided *p* values of < 0.05 were considered significant.

## Results

### Bench test

#### Filter

At an air flow rate of 60 L/min, the resistance of the DAR filter was measured at 3.3 cm H_2_O/L/s and the resistance of the Clear-Guard filter at 1.7 cm H_2_O/L/s. Since the FF-CPAP assembled with the DAR filter was used during the subsequent physiological evaluation and clinical study, by default we report below the results of the bench evaluation with this filter, unless otherwise stated. The full results of the bench evaluation, including those involving the Clear-Guard filter can be found in the Additional file 1.

#### Pressure and volumes

In static conditions on the Michigan test lung, when the filter was placed between the CPAP virtual valve and the test lung (representing the patient), the airway pressure measured inside the test lung with CPAP set at 6 or 10 cm H_2_O was not impacted by the presence or absence of a filter (difference of pressure < 0.1 cm H_2_O).

**Fig. 1:**
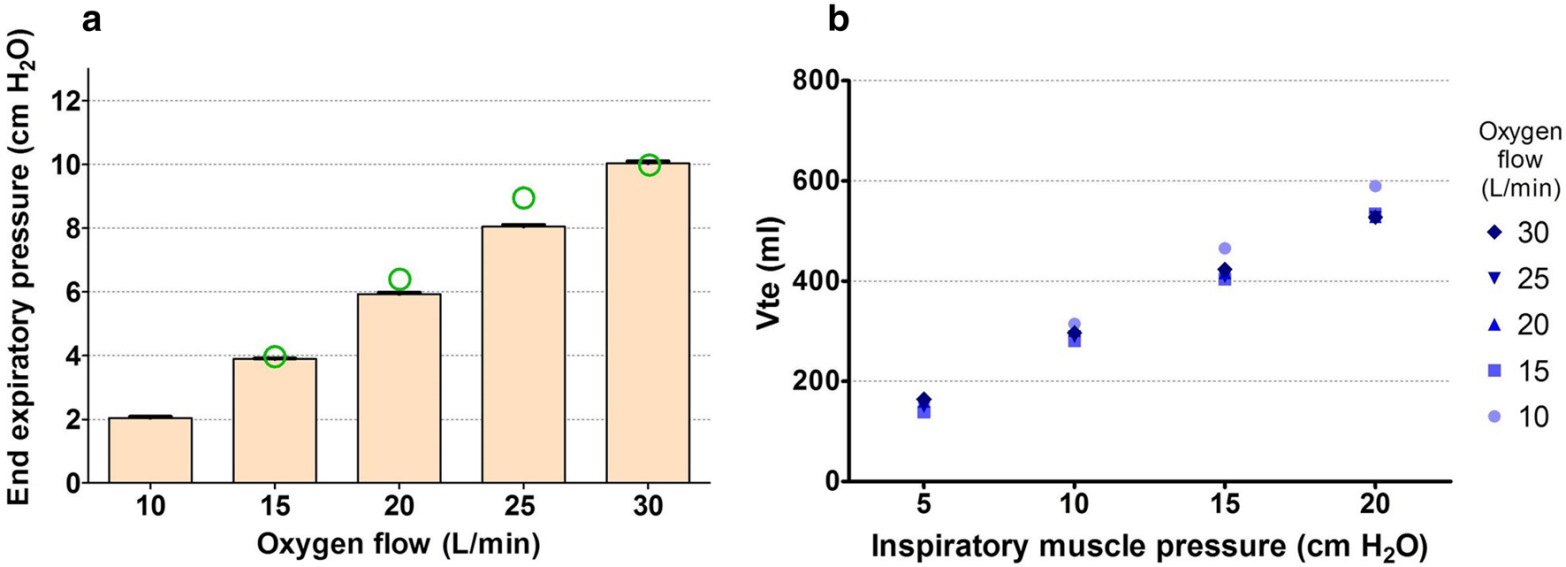
Bench study and physiological measurements. Changes in pressure and volume related to the oxygen flow rate. **a** Mean (bar chart) ± standard deviation (error bar) of end expiratory pressure recorded on the oro-nasal mask of Boussignac FF-CPAP assembled with the DAR filter (see text), obtained at five constant oxygen flow rates (10, 15, 20, 25, and 30 L/min) at four simulated inspiratory efforts (from weak to strong). Irrespective of the simulated respiratory effort, the observed end expiratory pressure on the mask was at 2, 4, 6, 8, and 10 cm H_2_O approximately, for oxygen flow of 10, 15, 20, 25, and 30 L/min, respectively. Green circles denote mean values recorded in four COVID-19 patients. **b** Tidal volume recorded for each combination of simulated inspiratory effort and constant oxygen flow. Tidal volume variations depended on simulated respiratory effort variations but not on oxygen flow (thus not on end expiratory pressure) variations

In dynamic conditions, FF-CPAP generated CPAP that increased upon increasing oxygen flow. Schematically, end expiratory pressure was 2, 4, 6, 8, and 10 cm H_2_O for oxygen flow of 10, 15, 20, 25, and 30 L/min, respectively, irrespective of simulated respiratory effort (Fig. 1a). The tidal volume increased with the increase in the simulated respiratory effort, but at a given respiratory effort, modifying CPAP level did not change the tidal volume (Fig. 1b).

Relative changes in spontaneous volume induced by the DAR and Clear-Guard filters, according to the different experimental conditions, are illustrated in Table 1 and Additional file 1: Table S1, respectively. For a similar effort, the additional filter slightly but significantly reduced spontaneous volume: 203 ± 85 mL vs. 236 ± 104 mL, *p* = 0.002. The tidal volume reduction was less with the clear-guard filter characterized by a lower resistance (see Additional file 1). Adding a filter to CPAP also significantly increased peak-to-peak airway pressure (P-P): 3.1 ± 1.4 cm H_2_O vs. 1.6 ± 0.5 cm H_2_O, *p* = 0.002.

**Table 1:** Bench study

Changes expressed in % of baseline value without filter		Low inspiratory effort (Pmus = −5 cm H_2_O)			Moderate inspiratory effort (Pmus = − 10 cm H_2_O)		
		∆ Volume	∆ WOB imposed	∆ WOB patient	∆ Volume	∆ WOB imposed	∆ WOB patient
Resistance 5 cm H_2_O/L/s	FF-CPAP 6 cm H_2_O	− 16.5%	+ 105.8%	+ 34.2%	− 15.5%	+ 98.3%	+ 29.9%
	FF-CPAP 10 cm H_2_O	− 15.3%	+ 64.8%	+ 32.5%	− 15.9%	+ 69.0%	+ 27.7%
Resistance 15 cm H_2_O/L/s	FF-CPAP 6 cm H_2_O	− 10.9%	+ 90.4%	+ 13.2%	− 11.3%	+ 95.9%	+ 8.5%
	FF-CPAP 10 cm H_2_O	− 9.4%	+ 51.7%	+ 14.5%	− 9.6%	+ 57.6%	+ 9.8%

Influence of the filter on tidal volume and work of breathing during low and moderate simulated inspiratory efforts

*Pmus* simulated muscle pressure, *∆ Volume* tidal volume variation induced by the filter as compared to baseline (without filter), *∆ WOBimposed* variation of work of breathing imposed by the CPAP induced by the filter as compared to baseline (without filter). WOBimposed was calculated from the airway pressure–volume loop. *∆ WOBpatient* variation of simulated patient’s work of breathing needed to maintain the tidal volume constant after having added the filter. WOBpatient was calculated from the muscle pressure–volume loop, *PEEP* positive end-expiratory pressure, *FF-CPAP* filter frugal continuous positive airway pressure (see text for definition)

#### Imposed work of breathing

Relative changes in WOBimposed induced by the filter, according to the different experimental conditions, are reported in Table 1, Additional file 1: Table S1 and Fig. S6. There was an increase in WOBimposed with the addition of the filter, which increased with the filter resistance, was similar whatever the level of effort (78 ± 21% vs. 80 ± 17% at efforts of 5 and 10 cm H_2_O, respectively, *p* = 0.571), but was mitigated by a higher level of CPAP (61 ± 7% vs. 98 ± 6% at CPAP of 10 and 6 cm H_2_O, respectively, *p* = 0.001). The increase in WOBimposed was lower with higher simulated resistances (74 ± 19% vs. 84 ± 18% at resistances of 15 and 5 cm H_2_O/L/s, respectively, *p* = 0.034).

#### Patient’s work of breathing

**Fig. 2:**
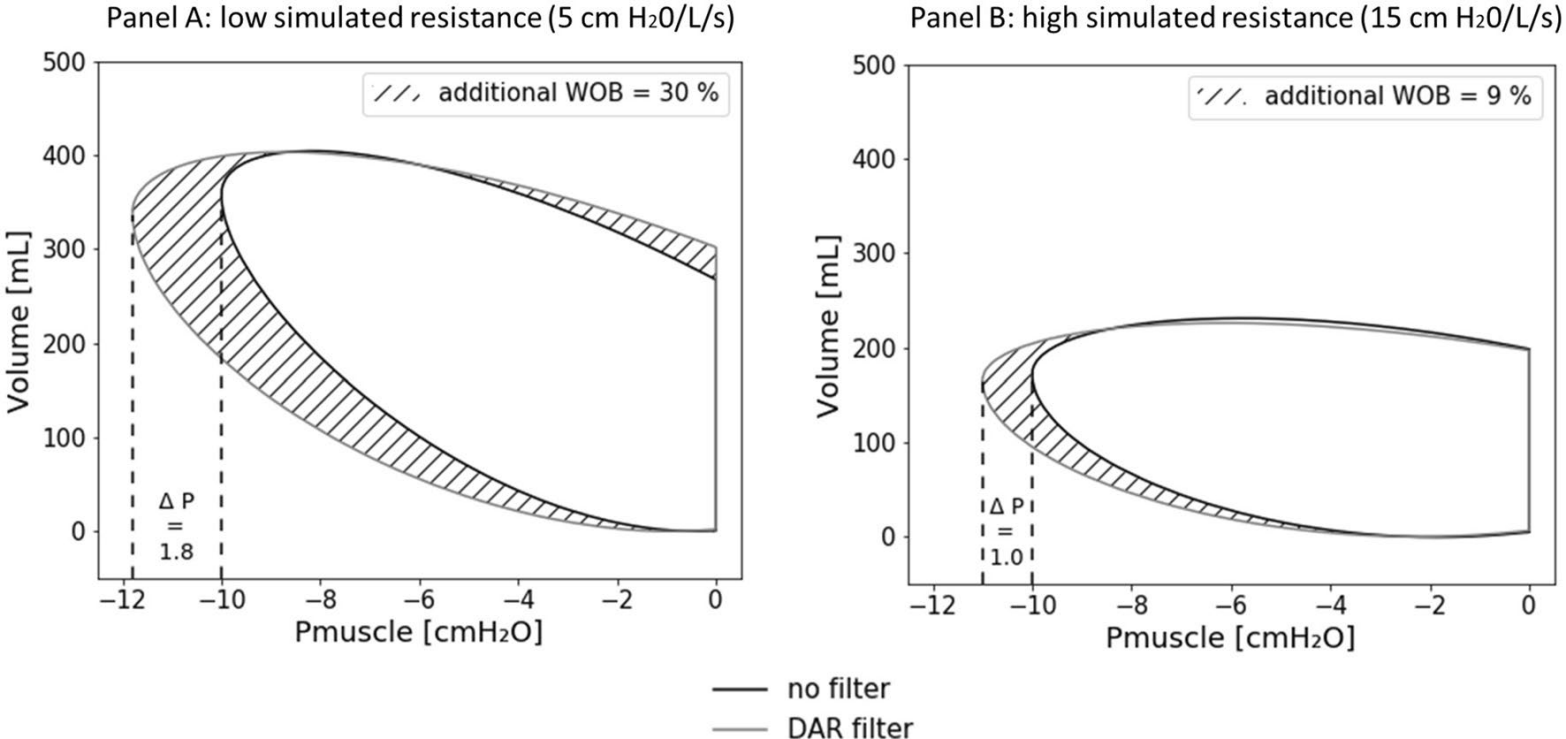
Bench study. The figure represents dynamic simulated muscle pressure (Pmus)–volume loops recorded with and without the addition of the DAR filter (see text). The change of tidal volume between configurations with and without filter was calculated. The pressure–volume loop with filter was obtained by increasing simulated patient effort to maintain Vt constant (same Vt than in the without filter configuration). The patient work of breathing (WOB patient) was defined as the trapezoidal numerical integration of the pressure–volume curve, which corresponds to the area under the curve. The shaded area represents the relative change in patient’s WOB induced by the filter to keep the Vt constant. ∆P represents the maximum change in muscle pressure between the two configurations. **a** Dynamic Pmus–volume loops obtained using the Boussignac valve with and without additional filter in the following conditions: moderate simulated effort (10 cm H_2_O), low simulated respiratory system resistance (5 cm H_2_O/L/s) and CPAP set at 6 cm H_2_O. **b** Dynamic Pmus–volume loops obtained using the Boussignac valve with and without additional filter in the following conditions: moderate simulated effort (10 cm H_2_O), high simulated respiratory system resistance (15 cm H_2_O/L/s) and CPAP set at 6 cm H_2_O

Relative changes in patient’s WOB induced by the filter, according to the different experimental conditions, are summarized in Table 1 and Additional file 1: Table S1. Dynamic pressure–volume loops were also reconstructed based on volume and muscular pressure recordings and are presented in Fig. 2 and Additional file 1: Fig. S7. The average additional patient’s WOB needed to sustain initial Vt without filter (∆WOBpatient) was 21 ± 10%. When the FF-CPAP was assembled with the Clear-Guard filter, the average ∆WOBpatient was noticeably lower (15 ± 7%, see Additional file 1^1^. The ∆WOBpatient was slightly higher for lower efforts (24 ± 10% vs*.* 19 ± 10% at Pmus of 5 and 10 cm H_2_O, respectively, *p* < 0.001), was not impacted by the level of CPAP (21 ± 11% vs*.* 21 ± 9% at PEEP of 6 and 10 cm H_2_O, respectively, *p* = 0.753), while it was mitigated by higher resistances (12 ± 2% vs. 31 ± 2% at resistances of 15 and 5 cm H_2_O/L/s, respectively, *p* < 0.001).

### Physiological measurements

In a pilot assessment of airway pressure in four COVID-AHRF patients (Additional file 1: Table S2, FF-CPAP achieved positive pressures similar to those obtained on the bench test (Fig. 1a).

### Training of the intermediate care units staff

All of the medical and paramedical staff got training on FF-CPAP using the dedicated video. Eight doctors and 16 nurses participated in the survey. All but three nurses reported that after watching the video they were able to mount a Boussignac FF-CPAP on a COVID-19 patient, and felt more comfortable with the procedure. The majority of doctors (88%) and nurses (75%) acknowledged that if they had not watched the video, they would have made mistakes. Beyond our hospital staff, thousands of medical staff worldwide (more than 55,000 learners from 146 countries) have benefited from this video-based training posted on MOOC.

### Clinical study

#### Patients

**Fig. 3:**
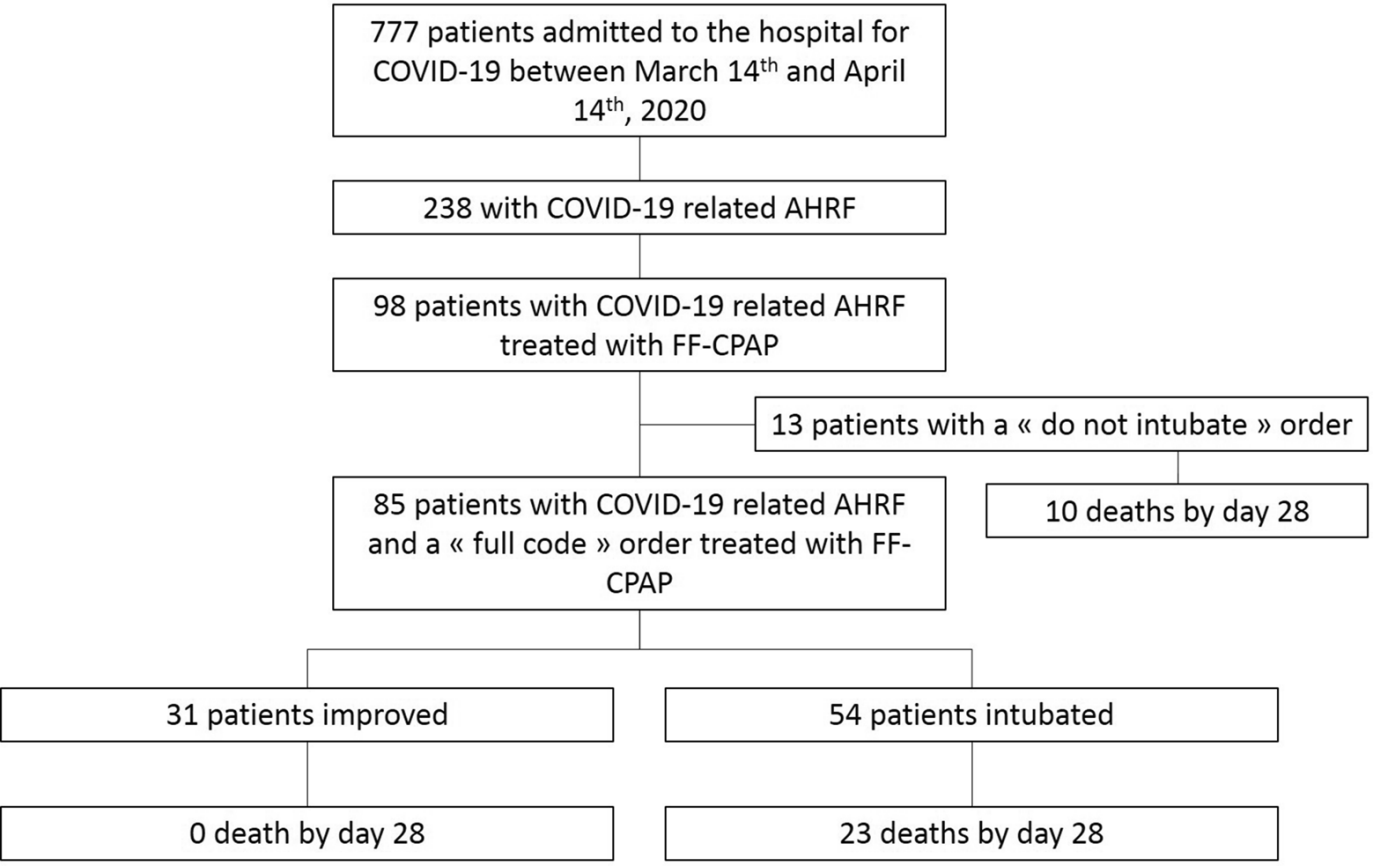
Clinical study. Flowchart of the study. COVID-19-related AHRF was defined as acute dyspnea (with a respiratory rate > 25 breaths/min and/or active contraction of accessory respiratory muscles), with escalating oxygen therapy ≥ 6 L/min with a non-rebreather facemask to maintain SpO_2_ > 90%, and new pulmonary infiltrates on chest X-rays [5] in a patient diagnosed with COVID-19. *FF-CPAP* filter frugal continuous positive airway pressure (see text for definition).

Between March 14th and April 14th, 2020, 98 COVID-AHRF patients used Boussignac FF-CPAP (Fig. 3). Thirteen were excluded as they had a “do not intubate” order, and 85 were included. SARS-CoV-2 pneumonia was confirmed by PCR from naso-pharyngeal swabs in 73 patients (86%) and by CT-scan in the remaining 12 patients (14%). Main baseline characteristics are shown in Table 2. All patients had an indication for ICU admission at baseline as they all experienced a COVID-AHRF with a median oxygen flow rate of 15 L/min (9–15 L/min) along with a tachypnea (median respiratory rate = 34 breaths/min [28–40 breaths/min]) and active contraction of accessory respiratory muscles (*n* = 85, 100%). Additionally, 36 patients (43%) exhibited predefined respiratory indications for intubation at the time of FF-CPAP initiation (Table 3): active contraction of accessory respiratory muscles (*n* = 36, 100%) and one of the following—respiratory rate above 40 breaths/min (*n* = 23, 64%) or acidosis with pH < 7.35 (*n* = 2, 6%) or SpO_2_ < 90% for more than 5 min despite an oxygen flow rate of at least 15 L/min (*n* = 17, 47%). Laboratory findings are reported in Additional file 1: Table S3.

**Table 2:** Baseline characteristics of patients on FF-CPAP

	All patients (*n* = 85)	FF-CPAP alone (*n* = 31)	FF-CPAP followed by intubation (*n* = 54)	*P* value
Demographic data				
Age, year	60 (50–68)	59 (46–64)	60 (53–70)	0.10
Female sex, *n* (%)	14 (16.5)	5 (16.1)	9 (16.7)	> 0.99
Body mass index, kg/m^2^	28.5 (24.5–31.1)	28.6 (24.7–30.7)	28.5 (24.4–31.9)	0.84
Medical history				
Current smoking, *n* (%)	5 (5.9)	2 (6.5)	3 (5.6)	> 0.99
Chronic respiratory disease, *n* (%)	8 (9.4)	2 (6.5)	6 (11.1)	0.71
Chronic cardiac disease, *n* (%)	8 (9.5)	4 (13.3)	4 (7.4)	0.45
Chronic renal disease, *n* (%)	9 (10.6)	3 (9.7)	6 (11.1)	> 0.99
Cancer, *n* (%)	9 (10.7)	2 (6.7)	7(13.0)	0.48
Treatment prior to hospital admission				
ACEI, *n* (%)	19 (22.4)	6 (19.4)	13 (24.1)	0.79
ARB, *n* (%)	14 (16.5)	3 (9.7)	11 (20.4)	0.24
Corticosteroids, *n* (%)	8 (9.4)	3 (9.7)	5 (9.3)	> 0.99
NSAI, *n* (%)	3 (3.5)	1 (3.2)	2 (3.7)	> 0.99
Symptoms potentially related to COVID-19				
Fever, *n* (%)	72 (84.7)	28 (90.3)	44 (81.5)	0.36
Cough, *n* (%)	64 (75.3)	24 (77.4)	40 (74.1)	0.80
Dyspnea, *n* (%)	67 (78.8)	27 (87.1)	40 (74.1)	0.18
Malaise, *n* (%)	5 (6.0)	2 (6.5)	3 (5.7)	> 0.99
Rhinorrhea, *n* (%)	5 (5.9)	1 (3.2)	4 (7.4)	0.65
Headache, *n* (%)	12 (14.1)	7 (22.6)	5 (9.3)	0.11
Diarrhea, *n* (%)	29 (34.1)	14 (45.2)	15 (27.8)	0.15
Vomiting, *n* (%)	10 (11.8)	6 (19.4)	4 (7.4)	0.16
Myalgia, *n* (%)	34 (40)	14 (45.2)	20 (37)	0.50
Chest pain, *n* (%)	4 (4.7)	1 (3.2)	3 (5.6)	> 0.99
Vital signs prior to FF-CPAP initiation				
Oxygen flow rate, L/min	15 (9–15)	12 (9–15)	15 (9–15)	0.11
Respiratory rate, breaths/min	34 (28–40)	30 (25–35)	35 (30–40)	0.009
Heart rate, beats/min	89 (79–100)	82 (75–95)	91 (85–100)	0.028
Mean blood pressure, mmHg	98 (89–106)	101 (93–104)	97 (88–107)	0.55
Arterial blood gases prior to FF-CPAP initiation				
pH	7.45 (7.42–7.47)	7.45 (7.42–7.48)	7.45 (7.42–7.47)	0.30
Bicarbonates, mmol/L	24.5 (22.3–26.3)	23.3 (21.9–26.1)	24.7 (23.6–26.8)	0.17
Lactate, mmol/L	1.3 (1.1–1.6)	1.5 (1.1–1.9)	1.3 (1.0–1.6)	0.37
PaO_2_, mmHg	73 (61–91)	71 (59–94)	74 (61–89)	> 0.99
PaCO_2_, mmHg	35 (31–38)	33 (30–38)	36 (33–39)	0.15
PaO_2_/FiO_2_^a^, mmHg	160 (115–258)	148 (111–248)	163 (115–277)	0.55
ROX index^b^	4.86 (3.67–6.37)	5.61 (4.54–7.21)	4.44 (3.55–5.70)	0.013
Percentage of involved parenchyma on CT-scan before FF-CPAP initiation (*n* = 65)				
≤ 50%, *n* (%)	27 (41.8)	9 (37.5)	18 (43.9)	0.79
> 50%, *n* (%)	38 (58.5)	15 (62.5)	23 (56.1)	0.79

^a^FiO_2_ was estimated as follows [5]: FiO_2_ (%) = 21 + [3 × oxygen flow rate (L/min)]

^b^ROX index was computed as follows: ROX index = (SpO_2_/FiO_2_)/respiratory rate, the FiO_2_ being estimated as described above

*FF-CPAP* filter frugal continuous positive airway pressure (see text for definition), *COPD* chronic obstructive pulmonary disease, *ACEI* angiotensin-converting enzyme inhibitor, *ARB* angiotensin II receptor blocker, *NSAI* non-steroidal anti-inflammatory drug within the 7 days before hospital admission

**Table 3:** FF-CPAP therapy conditions and patients’ outcome

	All patients (*n* = 85)	FF-CPAP alone (*n* = 31)	FF-CPAP followed by intubation (*n* = 54)	*P* value
Time between symptoms onset and FF-CPAP initiation, days	9 (7–13)	11 (7–13)	9 (7–12)	0.09
Time between hospital admission and FF-CPAP initiation, days	1 (0–4)	1 (0–4)	1 (0–4)	0.85
Predefined intubation criteria upon FF-CPAP initiation, *n* (%)	36 (42.6)	13 (41.9)	23 (42.6)	> 0.99
Vital signs during the first hour of FF-CPAP				
Oxygen flow rate, L/min	15 (15–15)	15 (15–15)	15 (15–30)	0.84
Respiratory rate, breaths/min	30 (25–38)	29 (25–32)	32 (24–42)	0.04
SpO_2_, %	96 (93–98)	96 (95–98)	95 (92–98)	0.05
Heart rate, beats/min	86 (72–101)	86 (72–96)	86 (72–107)	0.53
Treatment received during FF-CPAP therapy, *n* (%)				
Lopinavir/ritonavir	25 (29.8)	6 (19.4)	19 (35.8)	0.14
Hydroxychloroquine	51 (60.7)	16 (51.6)	35 (66)	0.25
Tocilizumab	12 (14.6)	7 (22.6)	5 (9.8)	0.20
Corticosteroids	8 (9.5)	4 (12.9)	4 (7.5)	0.46
Outcome				
FF-CPAP duration, days	2 (1–4)	4 (1.5–5.5)	2 (1–3)	0.02
Admission to the ICU, *n* (%)	68 (80)	14 (45.2)	54 (100)	< 0.001
28-day mortality, *n* (%)	23 (27.1)	0	23 (44.2)	< 0.001
28-day VFD, days	–	–	21 (0–26)	

*FF-CPAP* filter frugal continuous positive airway pressure (see text for definition), *VFD* ventilator-free days

### FF-CPAP therapy

**Fig. 4:**
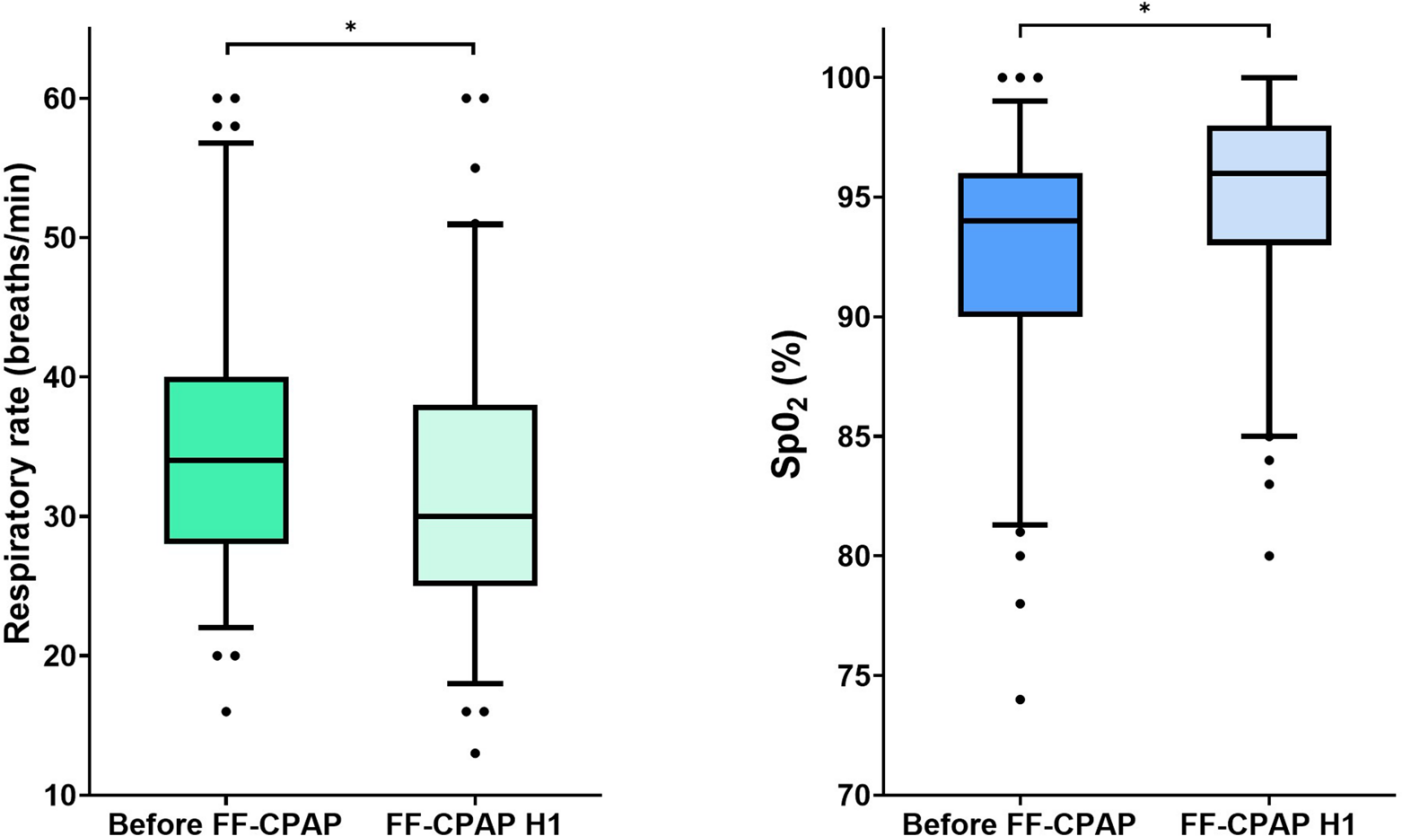
Clinical study. Effect of FF-CPAP on respiratory rate (**a**) and SpO_2_ (**b**). Respiratory rate and SpO_2_ were recorded before FF-CPAP initiation and then during the first hour of FF-CPAP therapy (FF-CPCP H1)

FF-CPAP was initiated 9 (7–13) days from COVID-19-related symptoms onset and one (0–4) day from hospital admission (Table 3). FF-CPAP was implemented in 72 (85%) patients in intermediate care units and in the remaining 13 (15%) in the ICU. The initiation of FF-CPAP was accompanied by a significant decrease in respiratory rate and a concomitant significant increase in SpO_2_ (Fig. 4). The median oxygen flow rate recorded with FF-CPAP was 15 (15–15) L/min for a median duration of 2 (1–4) days. FF-CPAP was never interrupted because of lack of training of nursing staff. Under FF-CPAP therapy, 31 patients (36%) improved and 17 patients (20%) remained in the intermediate care units without ICU admission. Among the 36 patients who exhibited predefined respiratory criteria for intubation prior to FF-CPAP initiation, 13 (36%) remained free from invasive ventilation, the remainder being intubated after two (1–3) days of FF-CPAP. Fifty-four patients (64%) required intubation within 28 days, after a median duration of FF-CPAP support of 2 (1–3) days. No cardiac arrest was observed prior to intubation in these patients.

**Fig. 5:**
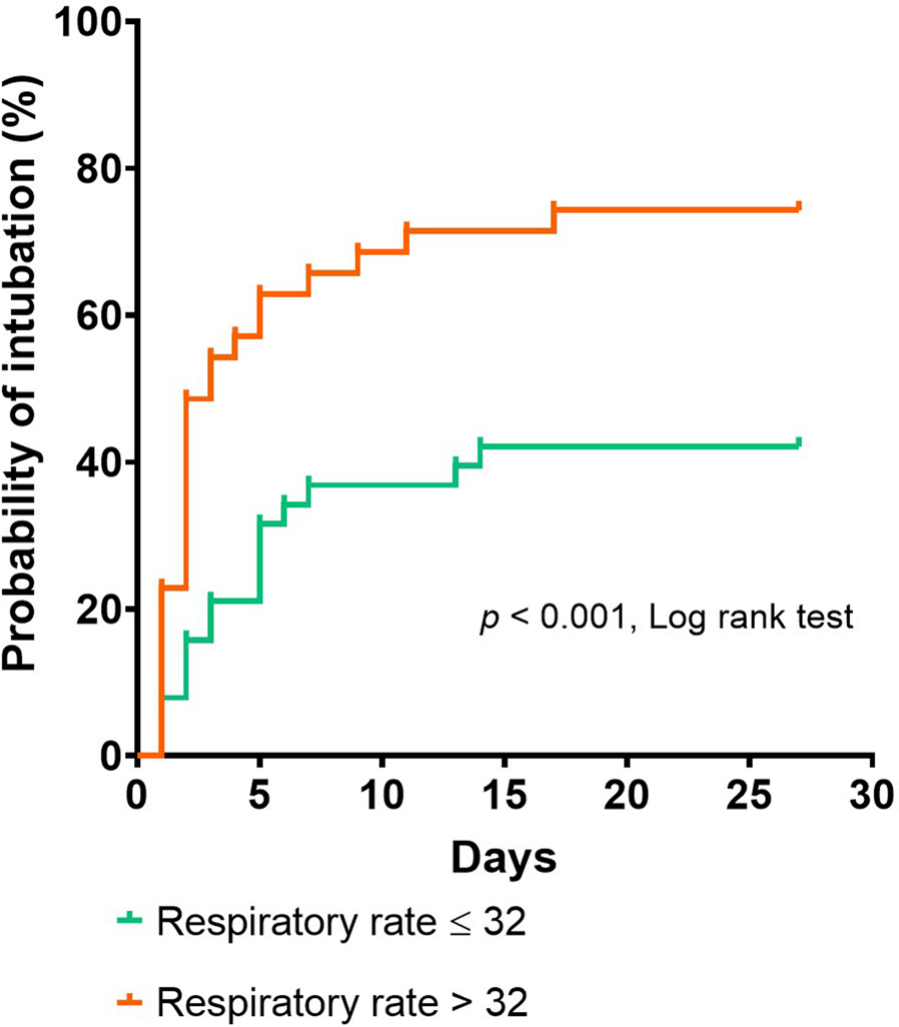
Clinical study. Kaplan–Meier estimate of the cumulative probability of intubation according to the respiratory rate prior to FF-CPAP initiation (higher or lower than 32 breaths/min)

Demographic data, co-morbidities, pre-hospitalization treatment, and symptoms type did not significantly differ between patients improving under FF-CPAP alone and those requiring intubation (Table 3). Patients who ultimately required intubation had a significantly higher respiratory rate at baseline than their counterparts (35 [30–40] vs*.* 30 [25–35] breaths/min, *p* = 0.009). A cut-off point of 32 breaths/min was identified as the most accurate to predict the need for intubation (see Additional file 1). Patients with a respiratory rate above 32 breaths/min at the time of FF-CPAP initiation had a significantly higher cumulative probability of intubation than their counterparts (*p* < 0.001 for log-rank test) (Fig. 5).

At day 28, all patients treated with FF-CPAP alone were still alive, whereas 23 (27%) of the 54 intubated patients died. Of the latter, the duration of FF-CPAP before intubation was not significantly different between patients who died and those who survived by day 28 (2 [0.5–5] vs. 1 [0.5–2] days, *p* = 0.12).

## Discussion

We herein report a comprehensive bench-to-bedside assessment of a frugal approach deployed to help manage a massive influx of COVID-AHRF patients during COVID-19 pandemic: the use in intermediate care units by non-ICU caregivers of a Boussignac valve on which we inserted an antimicrobial filter in order to limit the risk of viral aerosol dispersion (FF-CPAP). The results of our bench assessment could be summarized as follows: the filter did not affect the level of positive pressure actually delivered to the patient, but variably increased the effort required to maintain spontaneous tidal volume in simulating conditions. The theoretical increase in simulated patient’s effort depended on the resistive properties of the filter and was up to 34% when the simulated effort was low. The pressures obtained with the FF-CPAP on the bench test were retrieved in our physiological measurements in COVID-AHRF patients. We further showed that it was feasible to train the whole staff from intermediate care units for the use of FF-CPAP by the mean of an innovative massive training tool. Lastly, our clinical study provided the following main results: (1) the use of FF-CPAP to provide ventilatory support to COVID-AHRF patients in intermediate care units in the context of massive outbreak was feasible and accompanied by immediate improvement in oxygenation and signs of respiratory distress. (2) This strategy allowed some patients to improve without being admitted to the ICU and to gain a median of 2 days before intubation in others.

### The choice of CPAP to treat hypoxemia in COVID-19 patients

Hypoxemia is the main feature of COVID-AHRF [7]. However, providing adequate oxygen support to the most serious cases represents a challenge in COVID-19 pandemic. Early intubation may be inappropriate [7] and incurs rapid shortage of ICU beds [2]. Noninvasive ventilation (NIV) is not recommended in de novo AHRF [8]. Treating hypoxemia with NIV may be also simply impossible due to limited access to mechanical ventilators. High-flow nasal cannula may reduce the need for intubation in COVID-19 patients [9, 10], especially in case of ROX index below 5.4 [11], and does not result in a significant risk of viral aerosolization [12]. Its use in COVID-19 patients, however, requires specific devices whose availability and numbers were definitely insufficient in our center to face the massive influx of patients.

CPAP has been poorly assessed in de novo AHRF [13]. Encouraging data have been reported on the treatment of pneumonia-induced hypoxemia using CPAP [14, 15]. In the setting of COVID-19, CPAP could offer several advantages. First, it could be associated with limited viral aerosolization and medical staff contamination [16]. Secondly, it is a relevant solution to improve oxygenation and recruitment in COVID-19 patients [17, 18]. Thirdly, unlike bilevel NIV, increasing the assistance level during CPAP does not increase the tidal volume (as depicted in Fig. 1b) [19]. It is therefore less likely to enhance self-inflicted lung injury [20], provided it reduces the inspiratory effort. A preliminary report on 38 COVID-AHRF patients treated with CPAP suggested that it could spare intubation by day 14 [21]. Italian authors have also suggested using CPAP in COVID-19 patients with a “helmet” interface [22]. Most importantly, frugal devices providing CPAP outside ICU are available in sufficient quantities. We therefore built a frugal solution to treat COVID-19 patients by adding a bacterial filter to Boussignac CPAP [23].

### Bench assessment of the FF-CPAP

Our bench assessment of FF-CPAP regarding the pressure generated by different oxygen flow rates is consistent with a previous bench evaluation of Boussignac CPAP [24], and was further validated by our records in four patients. Our observations suggest that adding a filter may increase the effort needed to sustain ventilation, due to the resistive load of the FF-CPAP that is related to the filter’s resistance. Moreover, we cannot rule out the possibility that the filter resistance may have increased over time during prolonged use. Whether this resistive effect particularly marked with the filter used in the present clinical study may have mitigated the expected beneficial effect of the CPAP in COVID-19 patients and as a result affected the outcome is unknown. Nevertheless, whatever the filter resistance, humidification performance may also have an impact on patient comfort and tolerance especially when the FF-CPAP is used continuously with a high oxygen flow rate. Heat and moisture exchangers have been shown to adequately humidify inspired gases with the Boussignac CPAP [25]. Thus, among the multiple available devices [4], the best compromise between humidification performance and resistive properties might be seek to select a filter for assembling the FF-CPAP.

Interestingly, the higher the patient effort, the lower was the impact of the filter’s resistance. For clinical practice, these observations suggest that the negative impact of the filter on CPAP performances might be negligible in the most severe COVID-AHRF patients exhibiting strong respiratory effort and managed with at least 10 cmH_2_O of CPAP. On the contrary, the impact of the filter could be substantial in patient recovering from the acute phase. In that case, CPAP might better be stopped rather than progressively reduced when the patient improves.

### Implementation of intermediate care units and training

FF-CPAP therapy could be initiated in all patients thanks to its simplicity. Our video tutorial was efficient in providing non-ICU healthcare professionals with virtual training on FF-CPAP. This original training was an important part of our frugal approach during the pandemic to reach a large number of professionals who needed to know how to use FF-CPAP at distance and without trainers. Furthermore, its dissemination through a MOOC dedicated to COVID-19 crisis (https://www.fun-mooc.fr/courses/course-v1:UPEC+169003+archiveouvert/about) was unique in the field of critical care [26] and allowed the transferability of the FF-CPAP technique.

### FF-CPAP therapy

Within 1 month, FF-CPAP therapy could be initiated in up to 85 “full code” patients in our center. It is important to notice that our unselected population of COVID-AHRF patients were particularly critical with several markers of worse prognosis in terms of intubation and mortality rates (i.e., mostly men, advanced age, severe hypoxemia with a median oxygen flow rate of 15 L/min at inclusion) [27]. Especially, the median PaO_2_/FiO_2_ ratio upon FF-CPAP initiation was 160 mmHg. All patients had classical criteria for ICU admission and nearly a half exhibited predefined respiratory indications for intubation upon FF-CPAP initiation [6]. In these patients, FF-CPAP was associated with immediate improvement in oxygenation and signs of respiratory distress. It allowed some patients to overcome the critical period and to gain time for others. In fact, FF-CPAP support lasted 2 days (1–3 days) in median before intubation, even in patients exhibiting predefined criteria for intubation upon FF-CPAP initiation [6]. Overall, with FF-CPAP, ICU admission was spared in one-fifth of our population and delayed for many patients; interesting results to be considered in the current pandemic management. Of most, any solution to avoid ICU admission or to slowdown patient health deterioration for a few days or even hours is highly appreciated to reduce pressure on ICUs, provided it does not worsen the overall outcome. Regarding safety of using FF-CPAP in such severe cases out of the ICU, no cardiac arrest was observed prior to intubation. It is, however, worth mentioning that even though COVID-19 patients treated with FF-CPAP in intermediate care units were attended by non-ICU staff, continuous interactions with the ICU team was maintained, notably to discuss intubation indicators. Intubation was thus prompted in the absence of a rapid and clear response to treatment, meaning in case of persistence or occurrence of criteria for intubation. Increasing the risk of patient self-inflicted lung injury with FF-CPAP is a probability [20], but the lower mortality of intubated patients in our series, as compared with other reports, does not support such a hypothesis [28–30]. Besides, the duration of FF-CPAP prior to intubation was not different between patients who died by day 28 and survivors. Fifty-four (64%) patients were intubated in our series, which is consistent with previous reports. In a series of 49 patients with COVID-AHRF managed with comparable CPAP device, Alviset et al*.* reported an intubation rate of 62% [23]. In a series of patients receiving NIV for de novo AHRF, Thille et al*.* also reported an intubation rate of 62% when the PaO_2_/FiO_2_ ratio was below 200 mmHg [31]. Lastly, in critically ill COVID-19 patients, the reported intubation rate amounts to 70% [28–30]. A respiratory rate above 32 breaths/min before FF-CPAP initiation was associated with intubation in our series, hence the need to closely monitor such patients beforehand.

### Strengths and limitations

The bench-to-bedside assessment of a comprehensive frugal-based reasoning, incorporating a solution focused on the core need, an organizational dimension with an original training tool, is the main strength of our study. As for limitations, first, the clinical study is a retrospective study assessing data recorded in the medical chart during a massive outbreak, limiting the granularity of some data (e.g., the FF-CPAP therapy duration could be reported in days but not in hours) and the accessibility to others (e.g., the proportion of CPAP interruption due to lack of patient’s tolerance). However, because our local strategy to support COVID-AHRF patients with FF-CPAP involved systematic consultation with intensivists, we were able to easily identify every patient who received FF-CPAP therapy, thus limiting potential selection bias. Additionally, assessing as our main outcome the immediate effect of FF-CPAP support on respiratory symptoms and oxygenation limited the risk of potential confusion bias. Second, this is a single-center retrospective study without a control arm, conducted by an ICU team expert in handling noninvasive ventilatory support, which perhaps make our results not applicable in other centers. However, the international broadcasting of the training tool has the potential of ensuring homogeneity of FF-CPAP therapy initiation. Randomized clinical studies are urgently needed to prospectively assess its usefulness in this setting.

## Conclusion

Adding an antimicrobial filter to the Boussignac CPAP virtual valve (FF-CPAP) in order to limit contamination in the context of COVID-19 does not impact the level of positive pressure actually delivered to the patient, but may variably increase the resistive load, depending on the resistive properties of the filter. Our clinical results suggest that FF-CPAP could be an efficient frugal solution to provide a ventilatory support and improve oxygenation to numerous patients suffering from hypoxemic respiratory distress related to COVID-19 in intermediate care units.

## Supplementary Information

**Additional file 1.** Additional methods and results.

**Additional file 2.** e-Video: video tutorial to train non-ICU nurses and doctors to assemble and use FF-CPAP on COVID-AHRF patients.

**Additional file 3:** STROBE Statement—Checklist of items that should be included in reports of cohort studies.

## Data Availability

The datasets used and/or analyzed during the current study are available from the corresponding author on reasonable request.

## Abbreviations

AHRF: Acute hypoxemic respiratory failure
Clear-guard filter: Clear-Guard™ (Intersurgical^®^, Fontenay Sous Bois, France)
COVID-AHRF: COVID-19-related acute hypoxemic respiratory failure
CPAP: Continuous positive airway pressure
DAR filter: DAR™ adult–pediatric electrostatic filter HME small (Hygrobac S; Covidien, Medtronic, Parkway, MN, USA)
FF-CPAP: Filter frugal continuous positive airway pressure
MOOC: Massive open online course
Pmus: Muscle pressure
WOB: Work of breathing
WOBimposed: Work of breathing imposed by the device (CPAP or FF-CPAP)
WOBpatient: Patient’s work of breathing
