# Supplementary material for: Continuous positive airway pressure for respiratory support during COVID-19 pandemic: a frugal approach from bench to bedside
Source: Ann Intensive Care. 2021 Mar 2;11:38. doi: 10.1186/s13613-021-00828-2 (PMC7924341; doi:10.1186/s13613-021-00828-2)
Supplement: Supplementary file 1 — Additional file 1. Additional methods and results. [file 13613_2021_828_MOESM1_ESM.docx]

**ELECTRONIC SUPPLEMENTARY MATERIAL**

**Title**

**Continuous positive airway pressure for respiratory support during COVID-19 pandemic: a frugal approach from bench to bedside**

**List of Authors**

Guillaume CARTEAUX, Manuella PONS, François MORIN, Samuel TUFFET, Arnaud LESIMPLE, Bilal BADAT, Anne-Fleur HAUDEBOURG, François PERIER, Yvon DEPLANTE, Constance GUILLAUD, Frédéric SCHLEMMER, Elena FOIS, Nicolas MONGARDON, Mehdi KHELLAF, Karim JAFFAL, Camille DEGUILLARD, Philippe GRIMBERT, Raphaëlle HUGUET, Keyvan RAZAZI, Nicolas DE PROST, François TEMPLIER, François BELONCLE, Alain MERCAT, Laurent BROCHARD , Vincent AUDARD, Pascal LIM, Jean-Christophe RICHARD, Dominique SAVARY and Armand MEKONTSO DESSAP

# ADDITIONAL METHODS

## Description of the frugal CPAP:

Boussignac (Vygon, Ecouen, France) is a simple CPAP device, composed of an oro-nasal mask and a valve connected to an oxygen source (e-Figure 1). The low-cost plastic valve can generate a continuous positive airway pressure from a constant oxygen flow in a range that is usually available in the ward (10 to 30 L/min), without requiring any electric power source. The constant oxygen flow is split through the frugal CPAP valve into several narrow canals, creating high-velocity streams, which converge at the center of the chamber generating turbulence that results in a “virtual valve”. The level of continuous positive airway pressure generated in the frugal CPAP valve is determined by the oxygen flow rate. Thus, the operating principle of the frugal CPAP is simple and the device deployment does not require specific technical skills.

## Description of the “Filter Frugal CPAP”

As described above, the frugal CPAP works as an open system through a virtual valve. In order to protect the environment and the medical staff from any contamination from the patient’s airway, the principle of the “Filter Frugal CPAP” (FF-CPAP) is to add a filter with anti-viral properties, which acts as a “microbiological barrier” between the oro-nasal mask and the frugal CPAP valve. The connection requires a 22M-22F connector (e-Figure 2).

## Bench study

We conducted the entire bench assessment with two different filters characterized by different humidification and mechanical properties: the DAR^TM^ Adult − Pediatric Electrostatic Filter HME Small (Hygrobac S; Covidien, Medtronic, Parkway, MN, USA) and the Clear-Guard™ (Intersurgical®, Fontenay Sous Bois, France). The first one (subsequently named “DAR filter”) is a heat and moisture exchanger with high humidification performances [4], the second one is an electrostatic filter (subsequently named “Clear-Guard filter”) dedicated to CPAP with poor humidification performances but lower resistance. We measured the resistance of each filter at the following air flow rates: 30, 60, 90 and 120 L/min.

### Description of the dynamic assessment of the FF-CPAP

### Assessment of the pressure generated by the FF-CPAP at different oxygen flow rates.

#### Bench model

The first step of the bench evaluation was to assess the volume and pressure generated by the FF-CPAP at different oxygen flow rates. An oro-nasal mask (AcuCare non-vented mask, ResMed) was strapped to the face of a RespiSim® Manikin (IngMar Medical, Pittsburg, PA, USA) connected to an Active Servo Lung 5000 simulator (ASL5000®; IngMar Medical, Pittsburg, PA, USA). We carefully avoided leaks around the mask. The heat and moisture exchanger was an adult-pediatric electrostatic filter HME (DAR^TM^ Adult − Pediatric Electrostatic Filter HME Small, Medtronic, Parkway, MN, USA). The simulated respiratory mechanics was characterized by a respiratory system compliance of 50 cm H_2_O and a respiratory system resistance of 5 cm H_2_O/L/s; these settings were chosen based on initial reports on respiratory mechanics in COVID-19 patients [6].

Efficacy of the FF-CPAP was thus assessed by recording the pressure into oro-nasal mask at five constant oxygen flows: 10, 15, 20, 25, and 30 L/min while simulating four different inspiratory efforts using the following different simulated inspiratory muscle pressures: 5, 10, 15, and 20 cm H_2_O at a simulated respiratory rate of 20 cycles/min.

#### Recordings

Pressure into the oro-nasal mask was recorded using a differential pressure transducer TSD160D (Biopac Systems, Goleta, CA, USA) directly connected to the mask (e-Figure 3). Flow was recorded using AC137A-1 pneumotachograph (Biopac Systems, Goleta, CA, USA) inserted between the oro-nasal mask and the Boussignac valve. All signals were recorded at 2000 Hz using an analog/numeric data-acquisition system (MP150, Biopac systems, Goleta, CA, USA) and stored on a computer for subsequent analysis with AcqKnowledge software version 5.0 (Biopac systems, Goleta, CA, USA). End expiratory pressure was defined as the mean pressure recorded on the mask over the last 150 milliseconds of the expiratory time. The expired tidal volume was defined as the area under the negative flow curve. Every value was averaged over the last three consecutive respiratory cycles in each condition.

### Assessment of the impact of the FF-CPAP’s filter on the pressure transmitted to the patient.

#### Bench model

The Boussignac valve was connected to the airway opening of the ASL 5000 lung simulator. The volume, airway pressure and muscle pressure (Pmus) were recorded without and with the electrostatic filter in the following eight conditions: at two simulated effort (- 5 and -10 cm H_2_O of Pmus), two simulated resistances (5 and 15 cm H_2_O/L/sec) with a constant compliance of 50 mL/cm H_2_O and two levels of CPAP (6 and 10 cm H_2_O).

The decrease in volume (Delta Vt) induced by the filter and the maximum change in airway pressure between inspiration and expiration (expressed as Peak to Peak airway pressure (P-P)) were measured for each condition.

Dynamic pressure-volume loops were reconstructed based on volume and airway pressure recordings to calculate the work of breathing imposed by the device (WOBimposed, see e-figure 4). WOBimposed was measured as the trapezoidal numerical integration of the pressure volume curve, which corresponds to the area under the curve:

$$WOB_{IMPOSED}= \int Paw dV$$

In each condition, the relative change in WOBimposed induced by the filter (ΔWOBimposed) was calculated and expressed as a percentage of the WOBimposed without the filter.

Dynamic pressure-volume loops were also reconstructed based on volume and muscle pressure recordings, to calculate the theoretical increase in patient’s work of breathing required to maintain the tidal volume constant. It allowed estimating the patient’s work of breathing (WOB patient, see e-figure 5), and was defined as the trapezoidal numerical integration of the pressure volume curve, which corresponds to the area under the curve:

$$WOB_{PATIENT}= \int Pmus dV$$

Relative changes in patient’s work of breathing needed to maintain the tidal volume constant was calculated and expressed as a percentage of its value without the filter.

#### Recordings

The volume, airway pressure and Pmus were recorded by the ASL 5000 and sampled at 512 Hz during 60 seconds in the 16 different conditions.

## Physiological measurements

In four patients receiving ventilatory support with the selected FF-CPAP at four different oxygen flow rates (15, 20, 25, and 30 L/min), the pressure reaching the oro-nasal mask was recorded. A differential pressure transducer TSD160D (Biopac Systems, Goleta, CA, USA) was thus directly connected to the mask to record airway pressure at 2000 Hz using an analog/numeric data-acquisition system (MP150, Biopac systems, Goleta, CA, USA). The tracings were stored on a computer for subsequent analysis with AcqKnowledge software version 5.0 (Biopac systems, Goleta, CA, USA). End expiratory pressure was defined as the mean pressure recorded on the mask over the last 150 milliseconds of the expiratory time.

## Setup of intermediate care and training

The usefulness of the video tutorial used to train intermediate care medical and paramedical staff was assessed through a survey. Doctors and nurses of these units were asked to give a score to the following statements using a Likert scale model (Strongly disagree / Disagree / neutral / Agree / Strongly agree):

- After viewing the video "Boussignac CPAP and COVID-19", I am able to set up a Boussignac FF-CPAP on a COVID-19 patient.
- After viewing the video "Boussignac CPAP and COVID-19", I felt more comfortable to use Boussignac FF-CPAP on COVID-19 patients.
- If I had not watched the video "Boussignac CPAP and COVID-19", I would have made mistakes upon installing Boussignac FF-CPAP on COVID-19 patients.

# ADDITIONAL RESULTS

## Bench study

### Filter resistance

With air flow rates of 30, 60, 90 and 120 L/min, the resistance of the DAR filter was measured at 2.8, 3.3, 3.7 and 4.2 cm H_2_O/L/s respectively, and the resistance of the Clear-Guard filter at 1.6, 1.7, 2.0 and 2.2 cm H_2_O/L/s respectively.

### Pressure and volumes

Relative changes in spontaneous volume induced by the filter, according to the different experimental conditions, are illustrated in e-Table 1. For a similar effort, the additional filter significantly reduced spontaneous volume. The amount of tidal volume reduction was related to the resistive properties of the filter: 246 ± 97 ml (without) filter to 225 ± 86 ml (with the Clear-Guard filter); 236 ± 104 ml (without filter) to 203 ± 85 ml (with the DAR filter), *p* = 0.002 for both comparisons.

Adding a filter to CPAP also slightly but significantly increased peak-to-peak airway pressure (P-P): 2.5 ± 1.1 cm H_2_0 *vs.* 1.5 ± 0.6 cm H_2_O (Clear-Guard filter); and 3.1 ± 1.4 cm H_2_0 *vs.* 1.6 ± 0.5 cm H_2_O (DAR filter), *p* = 0.002 for both comparisons.

### Imposed work of breathing

Relative changes in WOBimposed induced by the filter, according to the different experimental conditions, are reported in e-Table 1. There was an increase in WOBimposed with the addition of the filter, which was similar whatever the level of effort (57 ± 13 % vs. 59 ± 12 % at efforts of 5 and 10 cmH_2_O, respectively, p = 0.454), but was mitigated by a higher level of CPAP (46 ± 4 % vs. 69 ± 4 % at CPAP of 10 and 6 cmH_2_O, respectively, p = 0.002). There was a trend towards a lower increase in WOBimposed with higher resistances (55 ± 12 % vs. 61 ± 12 % at resistances of 15 and 5 cmH_2_O/L/s, respectively, = 0.078).

### Patient’s work of breathing

Relative changes in patient’s WOB induced by the filter, according to the different experimental conditions, are summarized in Table 1. Dynamic pressure-volume loops were also reconstructed based on volume and muscular pressure recordings and are presented in Figure 2. The average additional patient’s WOB needed to sustain initial Vt without filter (∆WOBpatient) was 15 ± 7 %. The ∆WOBpatient was slightly higher for lower efforts (16 ± 7 % *vs.* 13 ± 6 % at Pmus of 5 and 10 cmH_2_O, respectively, p = 0.014), was not impacted by the level of CPAP (16 ± 8 % *vs.* 13 ± 6 % at PEEP of 6 and 10 cmH_2_O, respectively, p = 0.233), while it was mitigated by higher resistances (8 ± 2 % *vs.* 21 ± 4 % at resistances of 15 and 5 cmH_2_O/L/s, respectively, p = 0.009).

## Clinical study

Patients who ultimately required intubation had a significantly higher respiratory rate at baseline than their counterparts (35 [30-40] *vs.* 30 [25-35] breaths/min, *p* = 0.009). The area under ROC curve assessing the accuracy of respiratory rate at the time of FF-CPAP initiation in predicting intubation was 0.65 (95% CI, 0.55–0.80; *p* = 0.009). A cut-off point of 32 breaths/min on ROC curve was identified as the most accurate to predict the need for intubation, with 63% of sensitivity, 71% specificity, 80% positive predictive value, and 54% negative predictive value. Patients with a respiratory rate above 32 breaths/min at the time of FF-CPAP initiation had a significantly higher cumulative probability of intubation than their counterparts (*p* < 0.001 for log-rank test).

# FIGURE LEGEND

**e-Figure 1:**

Boussignac CPAP mounted on a manikin

**e-Figure 2:**

“Filter Frugal CPAP” (FF-CPAP) mounted on a manikin

**e-Figure 3:**

Experimental setup: a “Filter Frugal CPAP” with a pressure transducer connected to the oro-nasal mask and a pneumotachograph inserted between the mask and the Boussignac valve.

**e-Figure 4:**

Explicative figure representing the work of breathing imposed by the CPAP (WOB imposed). Airway pressure - volume curves of both settings (with and without filter) are plotted. The WOB imposed is defined as the trapezoidal numerical integration of the pressure volume curve, which corresponds to the area under the curve. The change of tidal volume between configurations with and without filter is displayed. The maximum change of airway pressure between inspiration and expiration (Peak to Peak airway pressure) called P-P is also illustrated.

**e-Figure 5:**

The figure represents dynamic muscle pressure (Pmus) - volume loops recorded with (FF-CPAP) and without an additional filter. The change of tidal volume between configurations with and without filter was calculated. The pressure volume loop with filter was obtained by increasing simulated patient effort to maintain the tidal volume constant. The patient’s work of breathing (WOB patient) was defined as the trapezoidal numerical integration of the pressure volume curve, which corresponds to the area under the curve. The maximum change of muscle pressure between the two configurations (called ∆P) was illustrated on the figure.

**e-Figure 6:**

WOB imposed (see e-Figure 4 for details) at low simulated inspiratory effort (Panel A) and moderate simulated inspiratory effort (Panel B) without filter (black solid line), with the DAR filter (grey solid line) and with the Clear-Guard filter (grey dashed line), with low simulated resistance.

**e-Figure 7:**

WOB patient (see e-Figure 5 for details) without (solid black line) and with (solid grey line) the filter at low (left panels) and high (right panels) simulated resistance. Top panels represent the WOB patient recorded without or with the DAR filter and bottom panels represent WOB patient recorded without or with the Clear-Guard filter.

**e-Figure 1**


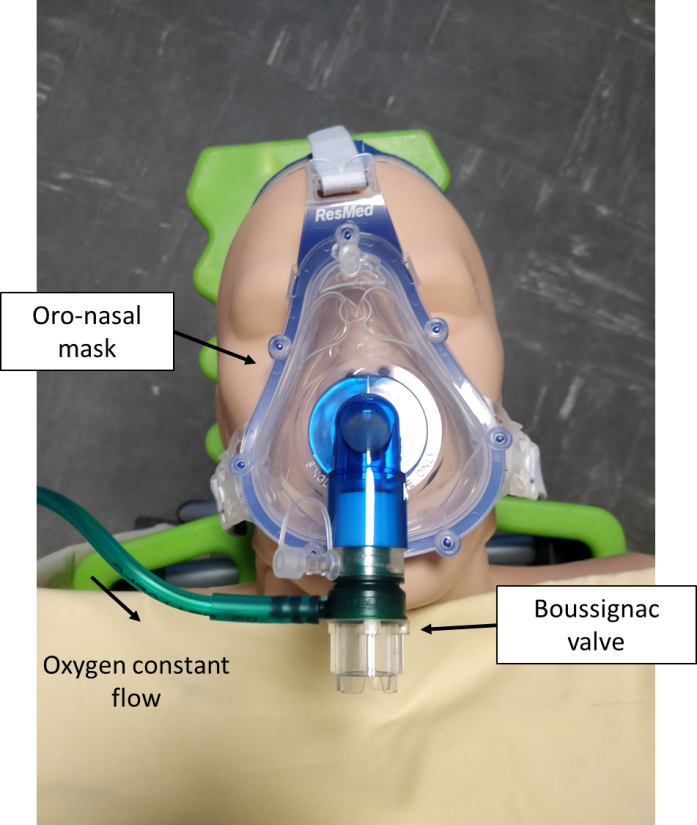


**e-Figure 2**


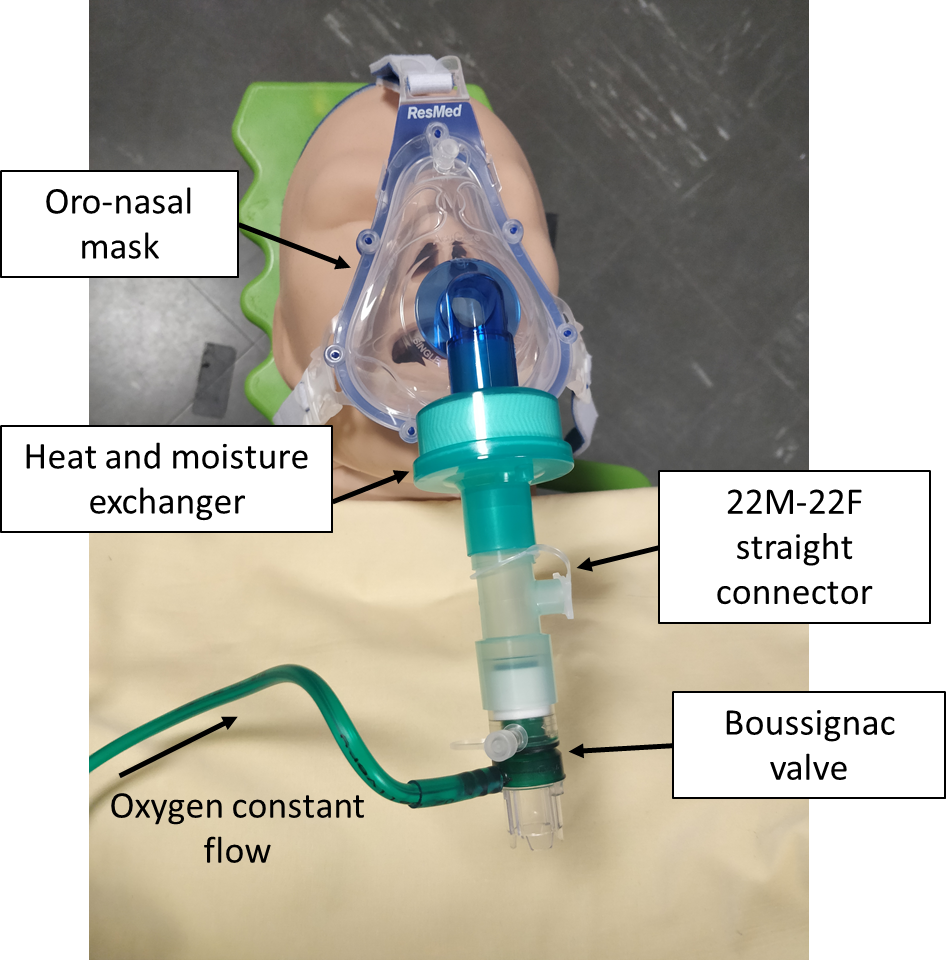


**e-Figure 3**


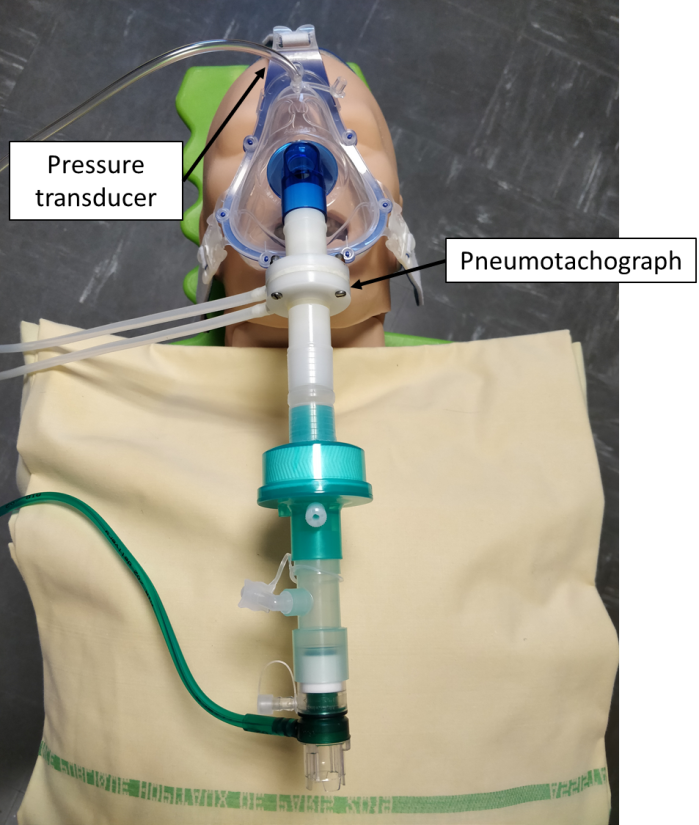


**e-Figure 4**


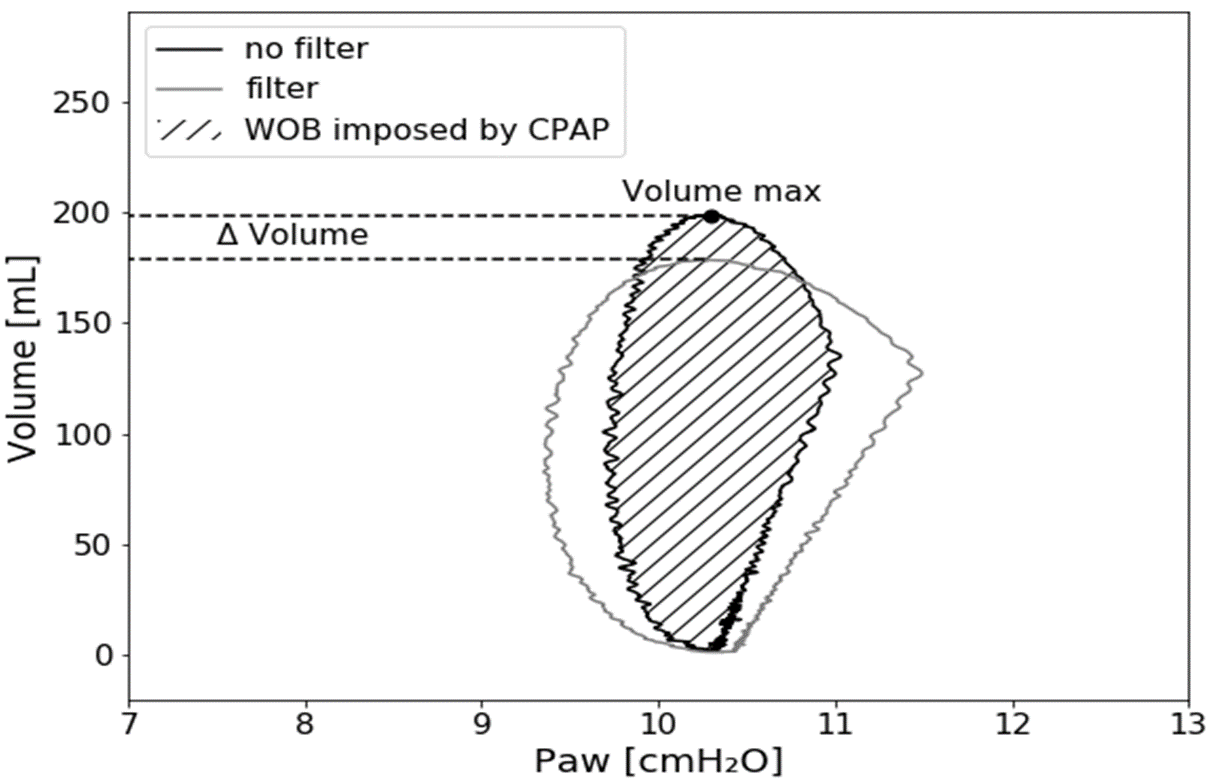


**e-Figure 5**


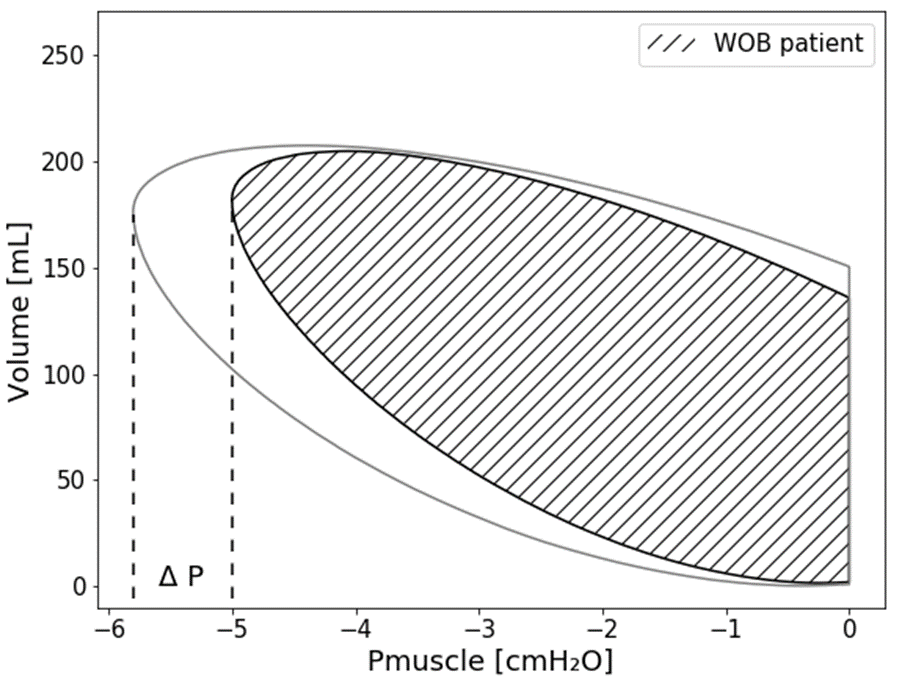


**e-Figure 6**


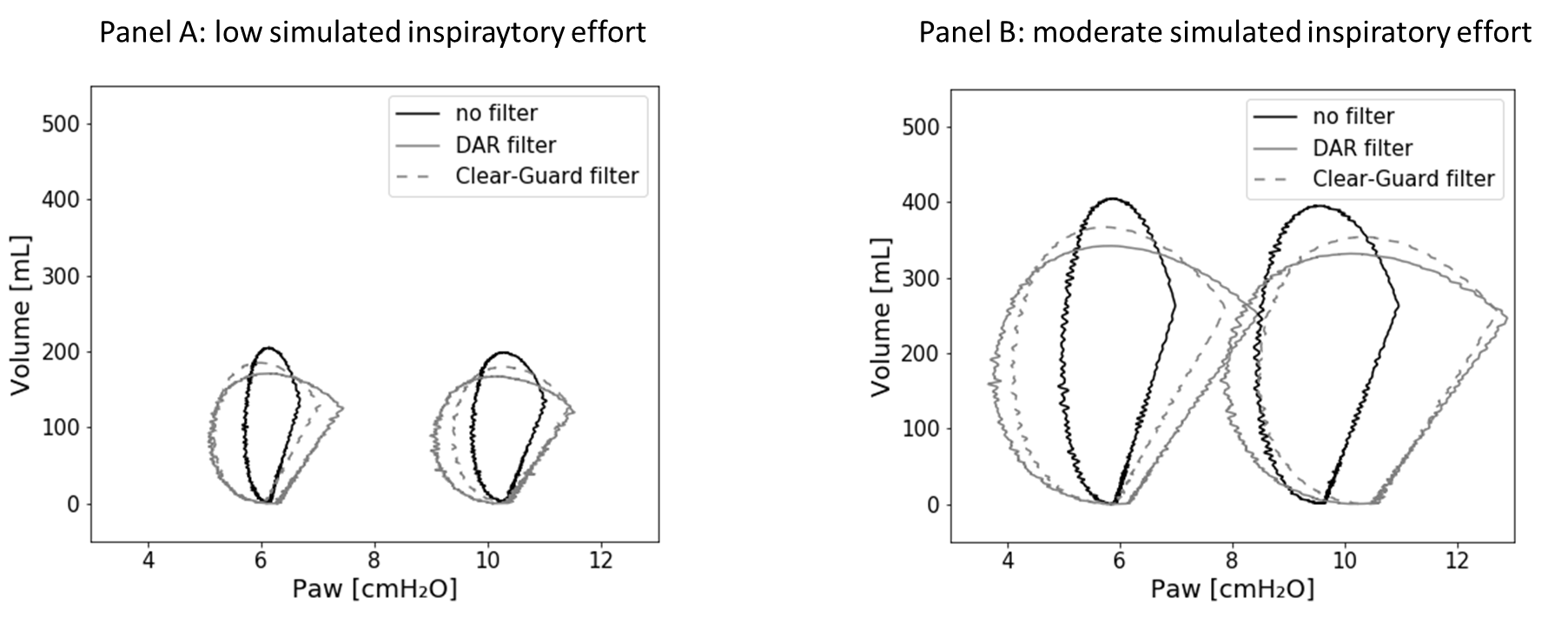


**e-Figure 7**


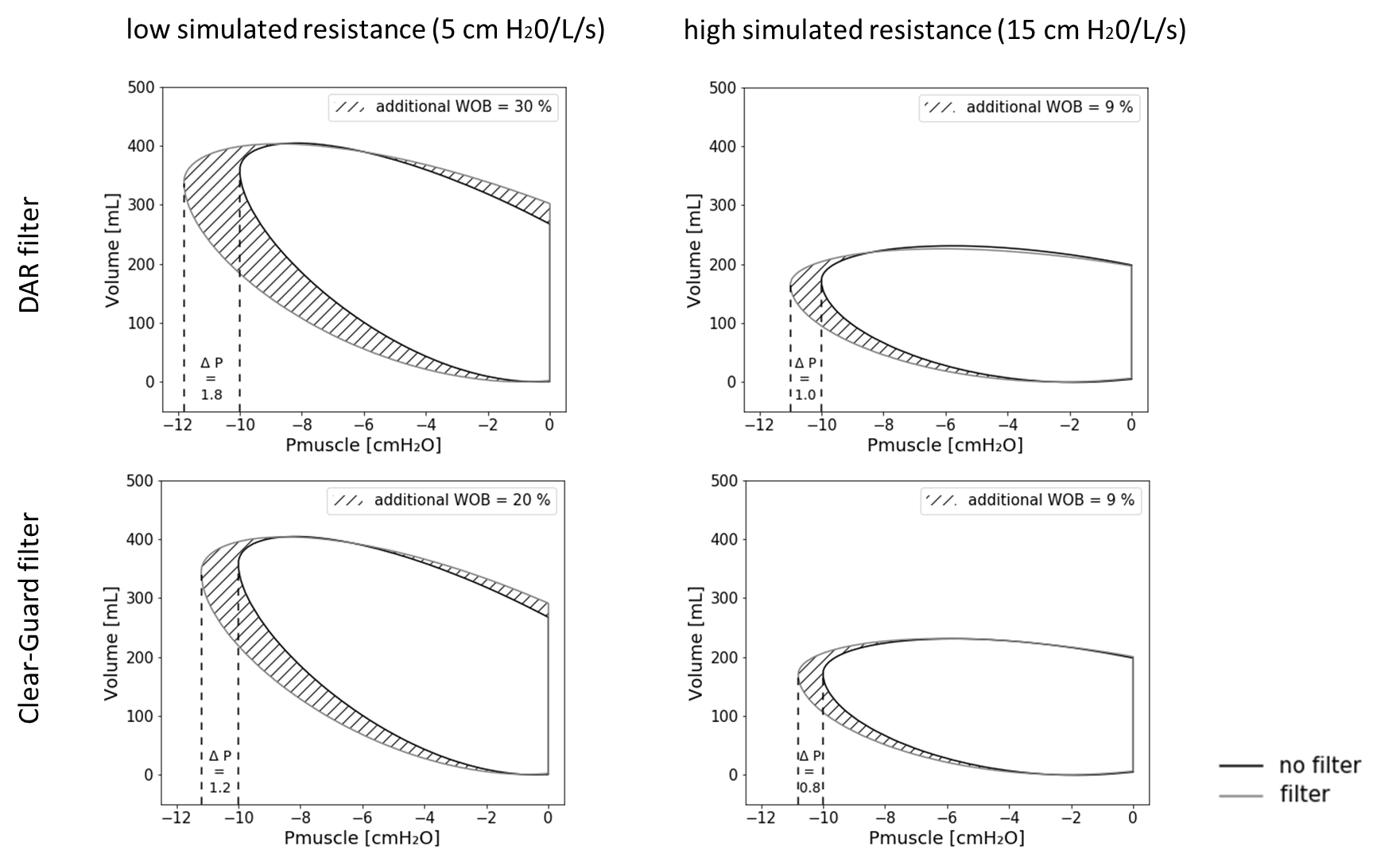


**e-Table 1. Bench study. Influence of the filters on tidal volume and work of breathing during low and moderate simulated inspiratory efforts**

|  | | | Low inspiratory effort  (Pmus = - 5 cm H_2_O) | | | Moderate inspiratory effort  (Pmus = - 10 cm H_2_O) | | |
| --- | --- | --- | --- | --- | --- | --- | --- | --- |
| Changes expressed in % of baseline value without filter | | | ∆ Volume | ∆ WOB imposed | ∆ WOB patient | ∆ Volume | ∆ WOB imposed | ∆ WOB patient |
| Resistance 5  cm H_2_O/L/s | FF-CPAP 6 cm H_2_O | DAR | - 16.5 % | + 105.8 % | + 34.2 % | - 15.5 % | + 98.3 % | + 29.9 % |
|  |  | Clear-Guard | - 9.7 % | + 73.5 % | + 27.7 % | - 9.4 % | + 70.5 % | + 20.1 % |
|  | FF-CPAP 10 cm H_2_O | DAR | - 15.3 % | + 64.8 % | + 32.5 % | - 15.9 % | + 69.0 % | + 27.7 % |
|  |  | Clear-Guard | - 9.8 % | + 45.2 % | + 18.3 % | - 10.6 % | + 51.3 % | + 18.7 % |
| Resisistance 15 cm H_2_O/L/s | FF-CPAP 6 cm H_2_O | DAR | - 10.9 % | + 90.4 % | + 13.2 % | - 11.3 % | + 95.9 % | + 8.5 % |
|  |  | Clear-Guard | - 6.6 % | + 61.3 % | + 9.4 % | - 6.7 % | + 69.3 % | + 8.6 % |
|  | FF-CPAP 10 cm H_2_O | DAR | - 9.4 % | + 51.7 % | + 14.5 % | - 9.6 % | + 57.6 % | + 9.8 % |
|  |  | Clear-Guard | - 5.9 % | + 46.3 % | + 10.1 % | - 6.5 % | + 44.1 % | + 5.7 % |

Pmus: simulated muscle pressure. ∆ Volume: tidal volume variation induced by the filter as compared to baseline (without filter). ∆ WOBimposed: variation of work of breathing imposed by the CPAP induced by the filter as compared to baseline (without filter). WOBimposed was calculated from the airway pressure - volume loop. ∆ WOBpatient : variation of simulated patient’s work of breathing needed to maintain the tidal volume constant after having added the filter. WOBpatient was calculated from the muscle pressure - volume loop. PEEP: Positive end-expiratory pressure. FF-CPAP: Filter Frugal Continuous Positive Airway Pressure (see text for definition). DAR: DAR^TM^ Adult − Pediatric Electrostatic Filter HME Small (Hygrobac S; Covidien, Medtronic, Parkway, MN, USA). Clear-Guard: Clear-Guard™ (Intersurgical®, Fontenay Sous Bois, France)

**e-Table 2**: **Patient's characteristics during the first 24 hours of FF-CPAP, and their outcome (physiological study)**

| Sex | Age (years) | BMI  (kg/m^2^) | Oxygen flow to  FF-CPAP (L/min) | Respiratory rate (cycles/min) | PaO_2_  (mmHg) | FF-CPAP duration (days) | Intubation |
| --- | --- | --- | --- | --- | --- | --- | --- |
| Male | 56 | 31 | 20 | 30 | 73 | 3 | yes |
| Male | 72 | 26 | 15 | 20 | 70 | 4 | yes |
| Male | 58 | 22 | 30 | 35 | 70 | 7 | yes |
| Female | 69 | 29 | 25 | 30 | 82 | 7 | no |

BMI: Body Mass Index, FF-CPAP: Filter Frugal Continuous Positive Airway Pressure

**e-Table 3. Laboratory findings (clinical study)**

|  | All patients  (n = 85) | FF-CPAP success  (n = 31) | FF-CPAP failure  (n = 54) | P value |
| --- | --- | --- | --- | --- |
| Creatinine, μmol/L | 79 (65-107) | 76 (66-92) | 82 (65-109) | 0.69 |
| Urea, mmol/L | 5.7 (4.1-9.0) | 5.7 (4.2-8.6) | 5.7 (3.9-11.1) | 0.83 |
| Creatine Kinase, U/L | 163 (68-542) | 93 (44-342) | 215 (95-624) | 0.10 |
| Lactate dehydrogenase, U/L | 398 (312-549) | 334 (263-539) | 424 (327-577) | 0.16 |
| Alanine aminotransferase, U/L | 43 (27-58) | 45 (24-63) | 42 (27-59) | 0.89 |
| Aspartate aminotransferase, U/L | 52 (41-80) | 45 (31-77) | 54 (42-80) | 0.24 |
| Alkaline phosphatase, U/L | 55 (46-93) | 62 (41-104) | 55 (47-87) | 0.79 |
| Gamma-glutamyl transférase, U/L | 74 (45-120) | 54 (42-107) | 82 (51-146) | 0.08 |
| Total bilirubin, mmol/L | 8.0 (6.0-11.0) | 8.5 (7.0-13.0) | 8.0 (5.7-11.0) | 0.16 |
| White blood cell count, x 10^9^/L | 7.5 (5.5-10.3) | 8.1 (5.5-10.3) | 7.5 (5.5-10.2) | 0.77 |
| Lymphocyte count, x 10^9^/L | 0.8 (0.5-1.1) | 0.8 (0.6-1.2) | 0.8 (0.5-1.0) | 0.57 |
| Neutrophil count, x 10^9^/L | 6.3 (4.1-9.1) | 7.7 (5.1-9.6) | 5.6 (3.7-9.1) | 0.38 |
| Platelet count, x 10^9^/L | 215 (155-276) | 259 (173-299) | 193 (147-252) | **0.04** |
| Hemoglobin, g/dL | 13.1 (12.0-14.4) | 13.3 (12.5-14.7) | 13.0 (11.7-14.3) | 0.30 |
| CRP, mg/L | 135 (86-238) | 120 (81-184) | 140 (86-295) | 0.28 |
| Procalcitonin, ng/mL | 0.4 (0.1-0.9) | 0.5 (0.1-1.0) | 0.3 (0.2-1.0) | 0.91 |

CRP, C reactive protein
